# Aggregation of montmorillonite and organic matter in aqueous media containing artificial seawater

**DOI:** 10.1186/1467-4866-10-2

**Published:** 2009-01-23

**Authors:** Yoko Furukawa, Janet L Watkins, Jinwook Kim, Kenneth J Curry, Richard H Bennett

**Affiliations:** 1 Naval Research Laboratory, Seafloor Sciences Branch, Stennis Space Center, Mississippi, USA; 2 Yonsei University, Department of Earth System Sciences, Seoul, South Korea; 3 University of Southern Mississippi, Department of Biological Sciences, Hattiesburg, Mississippi, USA; 4 SEAPROBE, Inc., Picayune, Mississippi, USA

## Abstract

**Background:**

The dispersion-aggregation behaviors of suspended colloids in rivers and estuaries are affected by the compositions of suspended materials (i.e., clay minerals vs. organic macromolecules) and salinity. Laboratory experiments were conducted to investigate the dispersion and aggregation mechanisms of suspended particles under simulated river and estuarine conditions. The average hydrodynamic diameters of suspended particles (representing degree of aggregation) and zeta potential (representing the electrokinetic properties of suspended colloids and aggregates) were determined for systems containing suspended montmorillonite, humic acid, and/or chitin at the circumneutral pH over a range of salinity (0 – 7.2 psu).

**Results:**

The montmorillonite-only system increased the degree of aggregation with salinity increase, as would be expected for suspended colloids whose dispersion-aggregation behavior is largely controlled by the surface electrostatic properties and van der Waals forces. When montmorillonite is combined with humic acid or chitin, the aggregation of montmorillonite was effectively inhibited. The surface interaction energy model calculations reveal that the steric repulsion, rather than the increase in electronegativity, is the primary cause for the inhibition of aggregation by the addition of humic acid or chitin.

**Conclusion:**

These results help explain the range of dispersion-aggregation behaviors observed in natural river and estuarine systems. It is postulated that the composition of suspended particles, specifically the availability of steric polymers such as those contained in humic acid, determine whether the river suspension is rapidly aggregated and settled or remains dispersed in suspension when it encounters increasingly saline environments of estuaries and oceans.

## Background

The dispersion-aggregation behavior of suspended colloids is important to the cycling of matter in rivers and estuaries. For example, the transport and fate of dissolved metal contaminants in rivers and estuarine environments are often directly determined by the potential co-aggregation and sedimentation along with the suspended colloids [1]. Excess nutrients that may be harmful to estuarine and coastal ecosystems and fisheries are sometimes removed through the natural processes of aggregation and sedimentation of suspended colloids [2]. The aggregation and dispersion of suspended colloids significantly alters the optical properties of coastal waters and thus a proper interpretation of remote sensing imagery requires the knowledge of the site-specific colloid aggregation-dispersion dynamics [3].

**Figure 1:**
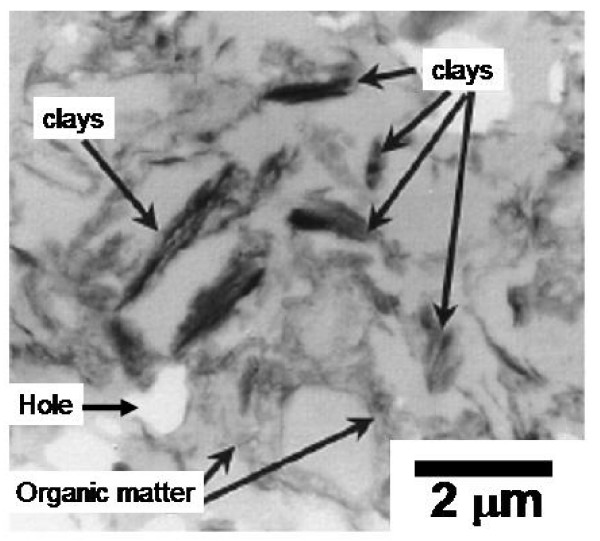
**Transmission electron micrograph (TEM) image of resin-embedded, ultrathin-section of fine-grained sediment from the Bay of St. Louis, Mississippi**. The fabric is characteristically porous, as the light grey color indicates pore space, now replaced by resin. Dark features represent clay mineral particles (domains) and heavy metal-stained organic matter. Bright areas are thin sectioning artifacts (i.e., holes after mineral grains were plucked out during thin-sectioning.)

Upon aggregation, river and estuarine colloids are settled to form bottom sediments. The sedimentary aggregates found in estuarine sediments are typically composed of open, porous organo-clay complexes (Figure 1). The essential components of these aggregates are colloidal-sized (< 2 μm) clay mineral particles and colloidal organic matter (organic detritus, living or dead cells, and their degradation and humification products) along with interstitial water and occasional free gas that fills the pore space [4,5]. In this report, we use the term "aggregates" to represent units or packets composed of many individual sedimentary colloids and particles. When the context necessitates, these units have sometimes been sub-classified into aggregates, agglomerates, fecal pellets, and flocs, based on the mechanical and/or physicochemical forces that hold the colloids and particles together [6].

It has been widely considered that the suspended colloids aggregate due to salinity increase when the river and estuarine waters are mixed with seawater in the vicinity of the river mouths. According to the popular Derjaguin-Landau-Verwey-Overbeek (DLVO) Theory, the interaction energy between two similarly charged suspended colloids is determined by the sum of the electrostatic repulsion between the electrical double layers (EDL) at the surface of both particles and van der Waals attraction between the particles. The DLVO theory elucidates the aggregation of suspended colloidal particles, such as hematite and latex colloids, due to increased ionic strength [7]. Increase in electrolyte concentrations allows EDL on the surface of charged colloids to diminish, allowing colloids to come closer and eventually succumb to van der Waals attraction.

**Figure 2:**
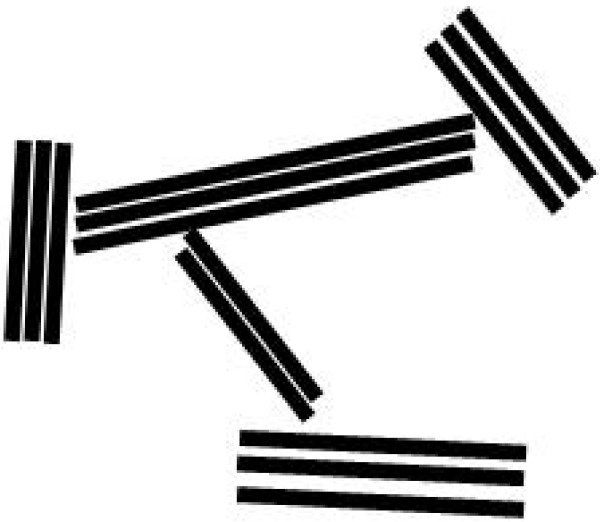
**Schematic drawings of a clay aggregate in a face-to-edge (F/E) orientation that is ubiquitous in unconsolidated fine-grained sediments**. A typical sedimentary clay colloid is composed of several layers of clay unit cells that have sheet-like morphology. (It appears as a stack of rods in this schematic 2D cross section.) Each colloid has large, negatively-charged basal planes (i.e., faces) and less negatively (or positively) charged edges.

The term "physicochemical flocs" has been used in the past to describe the initial formation of clay aggregates within water columns and in the immediate vicinity of sediment-water interface [4,8-10]. A similar physicochemical aggregation behavior has been also observed for iron oxides [11,12]. The previous clay studies argue that the open, typically "face-to-edge" association of clay colloids and particles in recent, unconsolidated fine-grained sediments (Figure 2) arises from the electrostatic attraction between negatively charged faces and positively, or at least less negatively, charged edges. The net surface charge of clay mineral particles is a result of two different types of surface charges: (i) permanently negative charge on the basal plane (i.e., "face") due to isomorphic substitution of Si by Al in Si-O_4 _tetrahedral sheets; and (ii) pH-dependent charge at the "edge" surfaces due to the reversible protonation and deprotonation of the surface hydroxyl groups [13]. The former is independent of pH and represents > 90% of the surface charge in the case of montmorillonite due to the platy morphology [14], whereas the latter is pH dependent. In low pH (i.e., high proton activity), the latter becomes less negative or even positive according to the reversible protonation and deprotonation reactions of silanol (Si-OH) and aluminol (Al-OH) surface groups. The edge surface charges of montmorillonite and kaolinite have been estimated from potentiometric titration to be very close to zero at circumneutral pH [15,16].

**Figure 3:**
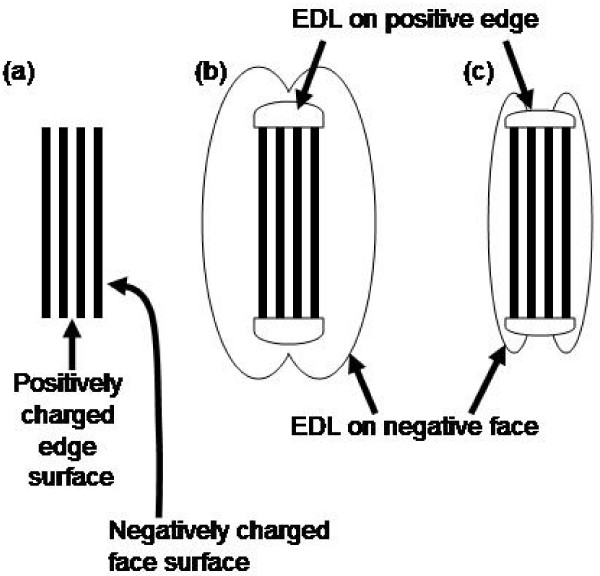
**Schematic representation of the electrical double layers (EDL) around a clay particle under low and high ionic strength conditions**. The pH < pH_pzc _is assumed. (a) A model clay particle (domain). (b) Clay particle suspended in a low ionic strength solution. The EDL is comparable to the particle thickness, and as a result, EDL on edge surfaces are masked by EDL on face surfaces. (c) Clay particle suspended in a high ionic strength solution. EDL is relatively thin, and EDL on edge surfaces are exposed. As a result, there is electrostatic attraction between oppositely charged face and edge surfaces. After Tombacz and Szekeres (2006).

The modified DLVO theory can explain how the attraction increases between negatively charged face surfaces and positively charged or neutral edge surfaces in solutions with higher concentrations of electrolytes (Figure 3) [17]. Essentially, the extended EDL for the negatively charged face surfaces can mask (or spill over on) the neutral or positive charge at edge surfaces in low salinity solutions. In this case the electrostatic force between the face and edge surfaces are repulsion due to their (apparent) negative charge. However in high salinity solutions, the "spill over" EDL is diminished and the edge surfaces are more exposed. Electrostatic repulsion is no longer strong, resulting in the face-to-edge arrangement of clay particles that are ubiquitous in fine-grained sediments. The rapid aggregation of river colloids upon salinity increase within estuarine environments, observed in several previous field and laboratory studies [18-21], may be explained by this mechanism.

However, numerous other studies report from environments in which there is little evidence of salinity-induced aggregation in river mouth and estuarine environments. [22-25]. Clearly, there are estuarine systems with suspended colloids in which DLVO-type interactions are not significant [4]. Previous studies show that colloidal suspension of clay minerals and iron oxides that also contain abundant organic matter (OM), especially humic acid (HA), resists aggregation under increased electrolyte concentrations [26-31]. In most cases, the resistance to aggregation has been qualitatively attributed to the adsorption of negatively charged organic polyanions on the clay edge surfaces and resulting strong negative charges on the edges [28,31-33]. The possibility of other repulsive forces (e.g., steric repulsion) has also been qualitatively alluded [30,33].

Thus, the purpose of this study is to investigate the effect of OM on the dispersion-aggregation behavior of river suspended colloids using the model organic matter (humic acid and purified chitin) and model clay mineral particles (< 1 μm montmorillonite, a common clay mineral type comprising the suite of clay minerals in marine and estuarine fine-grained sediments). The suspension concentrations typically found in OM-rich rivers of temperate regions were studied (i.e., 8 mg/L clay and up to 4.8 mg/L OM suspensions). The results were analyzed quantitatively using the surface interaction energy model based on the DLVO interactions between different model surfaces, in order to quantitatively elucidate the significance of non-DLVO interactions in the dispersion and aggregation of OM-rich colloidal suspensions in estuarine environments.

## Experimental Method

### Materials and preparation

Montmorillonite (Ward's Scientific, powdered bentonite, 46E0435), 2 g, was soaked in 1000 mL distilled water overnight, agitated, and settled for 7 hours and 22 minutes in a settling column. The supernatant in the upper 2.5 cm of the column containing the size fraction of < 1 μm was collected and saved. This process was repeated several times to collect enough material for an adequate suspension. The suspension concentration was checked by drying and weighing a 20 ml aliquot of the homogenized suspension, and then the remainder was adjusted by adding milli-Q water to yield a 16 mg/L stock suspension. After the addition of milli-Q water, the suspension was stirred for 1 minute before placing an aliquot in the sample cell for size analysis or electrokinetic analysis.

A chitin (Sigma, poly-[1→4]-β-N-acetyl-D-glucosamine, purified powder from crab shells, CAS 1398-61-4) sample of 16 mg was dissolved in 1 L milli-Q water and stirred for 1 hour. Visible sediment formed during the subsequent 1-hour settling was removed by filtering through 0.45 μm Supor membrane syringe filters. The chitin stock suspension was stored cold, used and discarded within 72 hours.

Humic acid (HA) (Aldrich, Humic acid sodium salt, 60% humic acid, CAS 68131-04-4) was dissolved in milli-Q water to yield 27 mg/L stock suspension (or 28 × 60% = 16 mg/L). This was stored cold, used and discarded within 72 hours. Before use, the HA suspension was filtered through 0.45 μm Supor membrane syringe filters to eliminate possible aggregates formed during storage.

These suspensions are combined so that the final suspension concentrations in experimental runs are 8 mg/L for montmorillonite, up to 4.8 mg/L for HA, and 4.8 mg/L for chitin. These suspension concentrations are within the typical values of material concentrations found in rivers. For example, waters from immediately above the upper estuary of the Pearl River in southern Mississippi has been characterized to contain 30 ± 20 mg/L total suspended solids (TSS) [34], with 21 weight % organic and 79 weight % inorganic materials (R. H. Stavn, unpublished data).

Artificial seawater (ASW) was prepared by dissolving 23.93 g NaCl, 4.01 g Na_2_SO_4_, 0.67 g KCl, 0.20 g NaHCO_3_, 10.83 g MgCl_2_·6H_2_O, and 1.52 g CaCl_2_·2H_2_O to 1 L milli-Q water (modified after [35]).

### Size analysis of suspended particles and aggregates

The size of suspended particles/colloids and aggregates was investigated by dynamic light scattering spectroscopy (DLS) using a Malvern Zetasizer nano-ZS equipped with MPT-2 titrator at 25°C. The details of DLS techniques can be found elsewhere [33,36]. Briefly, in DLS measurements, the temporal evolution of the intensity fluctuations of visible light that travels a known distance through an aqueous suspension is used to measure the translational diffusion coefficients of suspended colloids/particles. From the translational diffusion coefficient the average hydrodynamic diameter (*d*_*H*_) can be determined via the Stokes-Einstein equation. The actual calculations were done using Malvern's DTS^® ^software developed specifically for the Zetasizer.

In reality, the DLS measures the autocorrelation of the temporal fluctuations in the intensity of scattered light due to Brownian motion of the particles and colloids. The measured scatter is expressed as a function of time (i.e., correlation function). The translational diffusion coefficient is obtained from this function using the a fitting method called cumulant analysis [37,38]. The materials suspended in this study (i.e., montmorillonite particles/colloids, OM molecules, as well as their aggregates) are not spherical. Consequently, the *d*_*H *_values measured in this study are considered to be a measure of the *relative *sizes under given experimental conditions. In addition, it should also be noted that the materials vary in size due to the range of sizes in individual particles/colloids as well as the size variation due to aggregation. Consequently, the *d*_*H *_values reported in this study are the *average *hydrodynamic diameter. Whereas inversion methods (e.g., CONTIN) are often used in polydispersed systems to obtain the size distribution as well as *average d*_*H *_from the correlation function, the complexity of highly polydispersed natural systems with non-spherical particles make the application of inversion methods impractical. Thus the cumulant method was used in this study [37].

The *d*_*H *_values reported in this study are based on the size distribution by scatter intensity, rather than on the size distributions by volume or by the number of discrete particles [39]. The scattering intensity of a particle is proportional to the particle size to the sixth power. Consequently, the *d*_*H *_values reported here are a robust mean to compare the size differences of larger particles and aggregates, while being insensitive to the possible contributions from smaller particles that occupy less than 50% of the suspended material mass.

The DLS has been used successfully in previous laboratory studies to characterize the *d*_*H *_values of clay colloid and aggregate suspensions [28,33] as well as the that of HA macromolecules suspended/dissolved in aqueous media [37,40,41]. It should be noted that, even though the individual macromolecules of dissolved HA is very small (i.e., *d*_*H *_≈ 3 nm, [42]) approaching the lower resolution limit of the DLS analysis by Malvern Zetasizer nano ZS (i.e., *d*_*H *_> 0.6 nm, [39]), they usually take the form of aggregates, or supramolecules, in aqueous suspensions. The supramolecules are typically reported to be in the range of *d*_*H *_= 8 – 450 nm with the average *d*_*H *_values in the order of a few to several hundred nanometers [40,41].

The size analyses were conducted separately for montmorillonite-only suspension, chitin-only suspension, HA-only suspensions, montmorillonite plus chitin suspensions, and montmorillonite plus HA suspensions.

The effect of salinity on the sizes of suspended colloids/particles and aggregates were determined by tracking the time-dependent evolution of *d*_*H *_values following the mixing of ASW with the clay (+/- OM) suspensions using DSL. Prior to each analysis, the pH value was adjusted to ~7.2 with a small amount of 0.1 N NaOH or HCl. After mixing with ASW, the mixed aqueous solution was continuously stirred. Every 3 – 5 minutes, the mixed aqueous solution was introduced to the Zetasizer sample cell with the circulation system integrated into the MPT-2 titrator for the DLS analysis.

### Zeta potential analysis of suspended particles and aggregates

Zeta potential is an electrokinetic property of the EDL surrounding the particle. It is a potential at the slip plane that divides the diffuse layer into two regions: the inner diffuse layer where ions move with the particle movement, and outer diffuse layer where ions are still influenced by the particle due to long range forces but are not part of the coherent unit that moves with the particle. Even though the ζ-potential is defined as such and is strictly different from the surface potential, it is often used as a proxy for the surface potential as: (i) it represents the average electrokinetic behavior of the particles; and (ii) it can be determined experimentally unlike the surface potential.

The analysis of ζ-potential as a function of pH at a range of discrete salinity values were conducted by laser Doppler velocimetry (LDV) using a Malvern Zetasizer nano-ZS equipped with a MPT-2 titrator at 25°C. Each suspension sample, with an appropriate adjustment to pH and salinity, was loaded into a capillary cell with embedded electrodes at either of the two ends using the titrator. Suspended particles moved towards the electrode of the opposite charge when the potential was applied, and their velocity was measured and expressed in the unit field strength as their mobility. By knowing the physical properties of the suspension medium, the velocity can be converted to the ζ-potential using the Smolchowski equation [38]. The LDV techniques have been previously used to characterize the ζ-potentials of clays [43] and HA [44].

## Results

### Particle aggregation in montmorillonite plus OM systems

The average *d*_*H *_values of pure montmorillonite, HA, and chitin suspensions, without salinity increase, were determined by DLS to be 211 (± 50), 181 (± 23), and 269 (± 31) nm, respectively.

**Figure 4:**
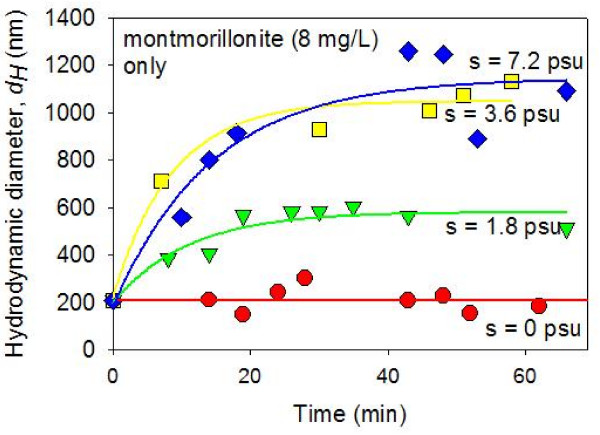
**Average hydrodynamic diameters (*dH*) of montmorillonite-only suspensions (8 mg/L) in constantly stirred solutions were measured as a function of time after mixing with artificial seawater (ASW) using dynamic light spectroscopy**. The pH was circumneutral. The results show a lack of aggregation in the zero salinity suspension. On the other hand, at elevated salinity values, (S = 1.8 – 7.2 psu), the colloidal particles rapidly aggregate during the initial ~15 minutes following the ASW mixing. In the S = 1.8 psu suspension, the aggregates reach the steady state average size of *dH *≈ 600 nm after 20 minutes. In the S = 3.6 and S = 7.2 psu suspensions, the aggregates become larger (*dH *≈ 1,100 nm) after the initial rapid aggregation.

Montmorillonite colloids in montmorillonite-only suspension form aggregates in constantly stirred solutions with elevated salinity (Figure 4). In zero salinity solutions, the value of *d*_*H *_remains at approximately 200 nm throughout the first 60 minutes after mixing with ASW. In systems with ASW (i.e., S = 1.8, 3.6, and 7.2 psu), the DLS analysis detected increase in the value of the average hydrodynamic diameter (*d*_*H*_) which indicates the formation of aggregates. This result agrees with the previous studies of laboratory kaolinite and montmorillonite aggregation in which the dispersion-aggregation properties of pure clay suspensions were found to be primarily determined by the solution ionic strengths (See Figure 3, also [29,45]). This is the behavior expected from suspensions whose dispersion-aggregation behaviors are primarily governed by the DLVO behaviors of competing electrostatic repulsion and van der Waals attraction.

**Figure 5:**
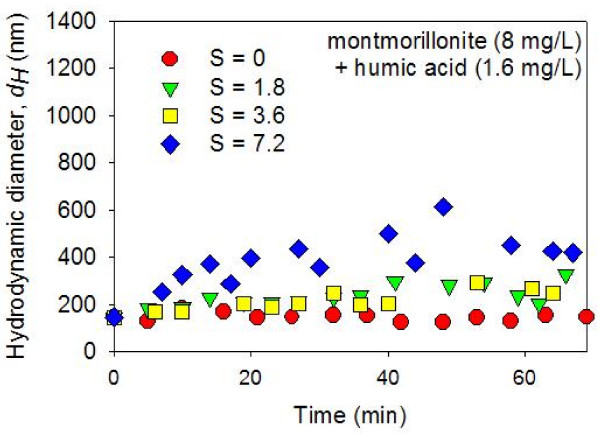
**Average hydrodynamic diameters (*dH*) of montmorillonite (8 mg/L) + HA (1.6 mg/L) suspensions in constantly stirred solutions were measured as a function of time after mixing with artificial seawater (ASW) using dynamic light spectroscopy**. The pH was circumneutral. The results show a lack of aggregation in the zero salinity suspension. In the suspensions with slightly elevated salinity (i.e., S = 1.8 and 3.6 psu), a slight increase in the average *dH *was detected toward the end of the time series observations (T ≈ 60 minutes). At the highest salinity value investigated (S = 7.2 psu), the colloidal particles gradually aggregated during the ~60 minutes following the ASW mixing.

**Figure 6:**
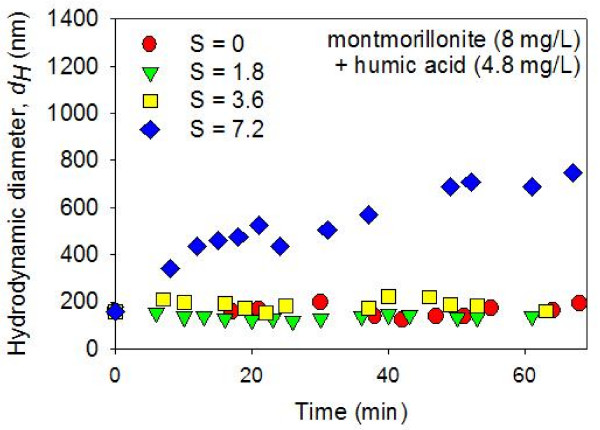
**Average hydrodynamic diameters (*dH*) of montmorillonite (8 mg/L) + HA (4.8 mg/L) suspensions in constantly stirred solutions were measured as a function of time after mixing with artificial seawater (ASW) using dynamic light spectroscopy**. The pH was circumneutral. The results show a lack of aggregation in the zero salinity suspension. In addition, no time-dependent aggregation was detected in suspensions with elevated salinity (i.e., S = 1.8 and 3.6 psu). At the highest salinity value investigated (S = 7.2 psu), the colloidal particles gradually aggregated during the ~60 minutes following the ASW mixing.

The colloidal suspensions in montmorillonite + HA systems did not aggregate as much as the montmorillonite-only suspensions upon mixing with ASW (Figures 5 and 6). With a small amount of HA (1.6 mg/L), the aggregation in S = 1.8 and 3.6 psu suspensions were trivial (Figure 5). With more HA (4.8 mg/L), no time-dependent aggregation was detected in S = 1.8 and 3.6 psu suspensions (Figure 6). With a higher salinity value (S = 7.2 psu), the mixed montmorillonite + HA suspensions went through some degree of time-dependent aggregation. However, the rate of aggregation was significantly slower than the rate of aggregation observed in the system with no HA (Figures 4, 5, 6). These results agree with the previous studies in which the salinity-induced aggregation of clays were hindered by the addition of HA [27]. It has been argued that the HA is adsorbed on the clay edge surfaces due to the surface complexation between clay aluminol and HA carboxyl groups [28,46]. This increases the dispersion by: (1) the increased negativity (or even charge reversal from positive to negative) at the clay edge surfaces (and thus increased electrostatic repulsion); and (2) steric repulsion due to polymeric components of HA [27,32,33].

**Figure 7:**
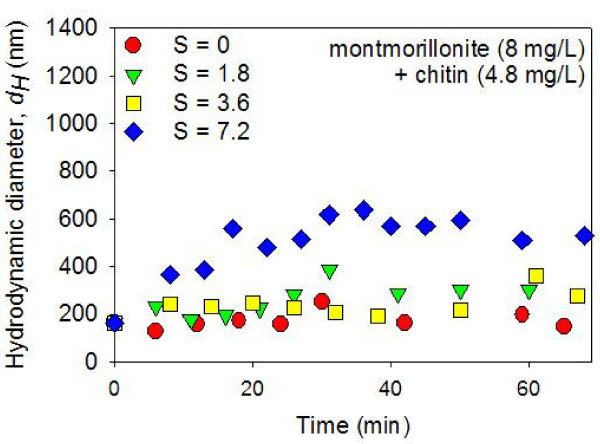
**Average hydrodynamic diameters (*dH*) of montmorillonite (8 mg/L) + chitin (4.8 mg/L) suspensions in constantly stirred solutions were measured as a function of time after mixing with artificial seawater (ASW) using dynamic light spectroscopy**. The pH was circumneutral. The results show a lack of aggregation in the zero salinity suspension. In addition, very little time-dependent aggregation was detected in suspensions with elevated salinity (i.e., S = 1.8 and 3.6 psu). At the highest salinity value investigated (S = 7.2 psu), the colloidal particles gradually aggregated during the ~60 minutes following the ASW mixing.

The colloidal suspensions in montmorillonite + chitin systems did not aggregate as much as the montmorillonite-only suspensions (Figures 7). With chitin (4.8 mg/L), very little, if any, time-dependent aggregation was detected in S = 1.8 and 3.6 psu suspensions. With a higher salinity value (S = 7.2 psu), the mixed montmorillonite + chitin suspensions went through some amount of time-dependent aggregation. However, the rate of aggregation was much slower than the rate of aggregation observed in the system with no chitin (Figures 4 and 7).

The results can be summarized as follows. (1) Montmorillonite-only suspension aggregates with increasing salinity, as expected from DLVO Theory: the system's dispersion-aggregation characteristics are primarily governed by the balance between electrostatic repulsion and van der Waals attraction. (2) The DLVO-driven aggregation of montmorillonite is inhibited by the addition of HA or chitin. The magnitude of inhibition is a function of salinity.

### Zeta potential of organic matter and aggregation behaviors

**Figure 8:**
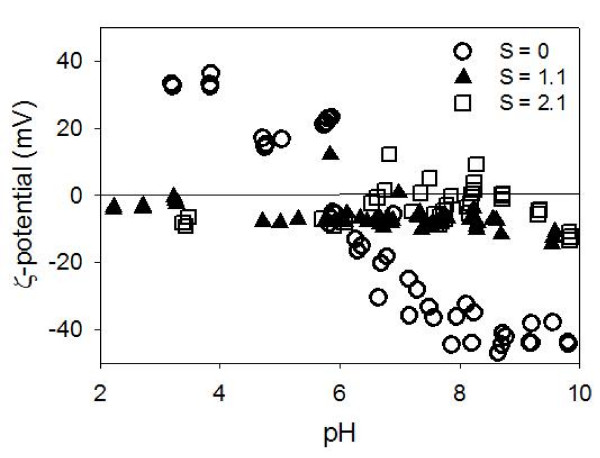
**The ζ-potential of chitin suspension as a function of pH at three discrete salinity values**. At zero salinity, z-potential is a function of pH, with the point of zero charge at approximately pH_pzc _= 5.5. However, when a small amount of electrolyte is present in the system, ζ-potential immediately becomes insensitive to pH, with its value very close to zero.

The ζ-potential of chitin as a function of pH at low salinity values (S ≈ 1.1 – 2.1) characterizes suspended chitin as particles with little electrostatic charges (Figure 8). This characterization comes from the near-zero ζ-potential values that are virtually independent of pH in aqueous solutions. The data in Figure 8 also reveal the pH-dependent surface charge in the zero salinity chitin suspension. This charge is derived from the protonation of the amino group at low pH and deprotonation of the hydroxyl group at high pH.

**Figure 9:**
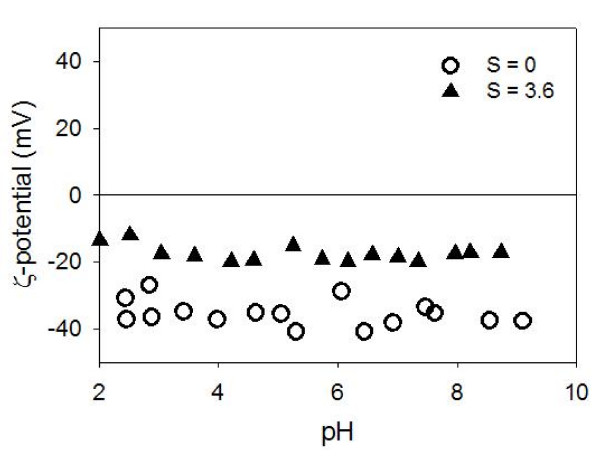
**The ζ-potential of humic acid (HA) suspension as a function of pH at two discrete salinity values**. At zero salinity, ζ-potential is independent of pH and highly negative, indicative of electrostatically stable colloidal suspension. At a higher salinity, ζ-potential is less negative but still at Φ_Z _≈ -20 mV.

The ζ-potential of HA molecules as a function of pH at two different salinity values characterizes humic acid as negatively charged colloids (Figure 9). This negative charge is primarily due to phenolic and carboxylic functional groups. The increase in salinity decreases the negative ζ-potential value to a certain degree, as would be expected from the availability of counter-ions.

**Figure 10:**
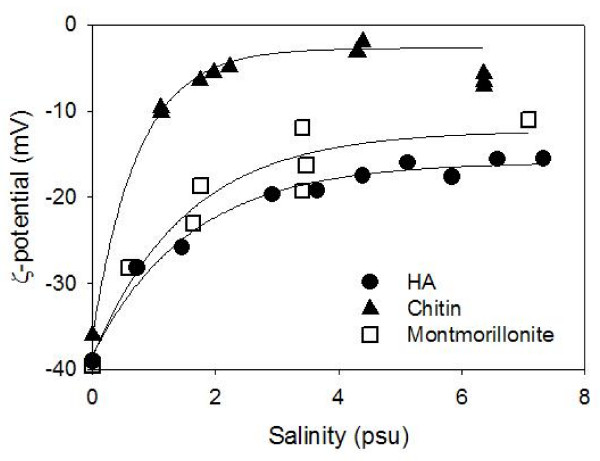
**The ζ-potential of humic acid (HA), chitin and montmorillonite suspensions at circumneutral pH (7 < pH < 7.5) as a function of salinity**. Note that the value for montmorillonite are averages over different surfaces including permanently negative face surfaces and less negative (and neutral or even positive in low pH) edge surfaces.

The ζ-potential values of pure montmorillonite, HA, and chitin suspensions as a function of salinity (Figure 10) reveal that the ζ-potential is a very strong function of salinity in very low salinity suspensions (S ≈ 0 – 2 psu) with less salinity dependency at higher salinity (S > 2 psu).

## Discussion

Previous studies of clay-OM interactions in the context of dispersion and aggregation mostly focused on the role of OM in modifying the surface electrostatic properties of the clays [27-29,33,45]. They showed that the adsorption of OM on clay surfaces, primarily through surface complexation between clay edge aluminol and OM's acidic functional groups, increased the electronegativity of the clay surfaces, resulting in increased electrostatic repulsion. The presence of steric repulsion has been qualitatively mentioned, but has not been explicitly quantified. We used the surface interaction energy model to calculate the contribution of electrostatic repulsion and van der Waals attraction to the observed dispersion-aggregation behaviors, in order to estimate quantitatively the magnitude of other repulsive forces (e.g., steric repulsion).

Particle interaction in a system that contains montmorillonite particles with OM adsorption on edge surfaces is controlled partly by electrostatic and Lifshitz-van der Waals contributions, as well as steric repulsion of OM polymers [29]. The relative magnitudes of these contributions determine whether the particles aggregate or remain dispersed and suspended in solution.

One mechanism for the montmorillonite dispersion is the electrostatic repulsion [29]. The bare montmorillonite edges are nearly neutrally charged in contrast to the negatively charged face surfaces. This surface charge difference leads to the likelihood of face-to-edge aggregation upon increased salinity (and thus diminished "spillover" EDL) (Figure 3). However, once HA molecules are adsorbed onto the edge surfaces, the HA-covered surface will become negatively charged, even in solutions with modest electrolyte concentrations. This would prevent the edge-to-face aggregation from occurring easily. Chitin does not have the same electrostatic effect as it is very close to neutrally charged in suspensions with any amount of electrolytes (Figures 8 and 10). If no other forces (i.e., steric repulsion) are present, these electrostatic repulsive forces need to overcome Lifshitz-van der Waals attractive forces if the particles are to remain in suspension as we observed in this study.

The electrostatic interaction energy between phases *i *and *j *in aqueous medium per unit area, $V_{ij}^{EL}$, is calculated using the Hogg, Healy and Fuerstenau (HHF) model [47], based on the assumption of constant, moderate surface potentials on two infinite flat planes. It should be noted that this model is appropriate when surface-to-surface distance *H *satisfies *H *≥ 10 nm.

(1) $$V_{ij}^{EL}=\frac{\varepsilon \varepsilon_{0}\kappa }{8\pi }\left[\left(\psi_{0i}^{2}+\psi_{0j}^{2}\right)\left(1-\coth\kappa H\right)+2\psi_{0i}\psi_{0j}\cos ech\kappa H\right]$$

[48]. Here ε is the dielectric constant of the medium (ε = 78.4 for aqueous medium at 25°C), ε_0 _is the permittivity in vacuum, κ is the reciprocal Debye length which is a function of ionic strength *I *(i.e., $\kappa =\frac{\sqrt{I}}{0.3082}$ (nm^-1^)), and subscripts (*i*, *j*) represent different surfaces (e.g., *f *for montmorillonite face, *c *for chitin-covered edge, and *ha *for HA-covered edge). The surface potential of phase *i*, ψ_0*i*_, can be reasonably approximated by the zeta potential, ψ_*zi *_(mV).

In the following calculations, the ζ-potentials of chitin- and HA-covered edges, ψ_*zc *_and ψ_*zha*_, were estimated to be equal to that of free-suspending chitin and HA that were determined experimentally for circumneutral pH as described above and shown in Figure 10 as a function of salinity. The data were modeled using the exponential rise function as functions of salinity (*S*, psu) as follows:

(2) ψ_*zc *_= -36.0 + 33.3(1-*e*^-1.36*S*^) (mV)

(3) ψ_*zha *_= -38.2 + 22.3(1-*e*^-0.626*S*^) (mV)

The ζ-potential of the montmorillonite face surface, ψ_*zf*_, was assumed to be equal to the bulk ζ-potential of the montmorillonite suspension because the edge surface represents only a small fraction (< 1%) of the net surface area [48]. It is reported in Figure 10 for circumneutral pH, and the data were modeled as follows:

(4) ψ_*zf *_= -38.5 + 26.3(1-*e*^-0.655*S*^) (mV)

The Lifshitz-van der Waals contribution, $V_{ij}^{LW}$, was calculated using the following equation [48]:

(5) $$V_{ij}^{LW}=-\frac{A_{ij}}{12\pi H^{2}}$$

where *A*_*ij *_is the Hamaker constant corresponding to the van der Waals interaction between phases *i *and *j *in aqueous medium. In this case, we assume that the van der Waals interaction between the montmorillonite face and the OM-coated edge surfaces is similar to that between the montmorillonite face and the bare edge, and thus the value of *A*_*ij *_was taken from the literature to be *A*_*fe *_= 7.3 × 10^-21 ^J [48].

**Figure 11:**
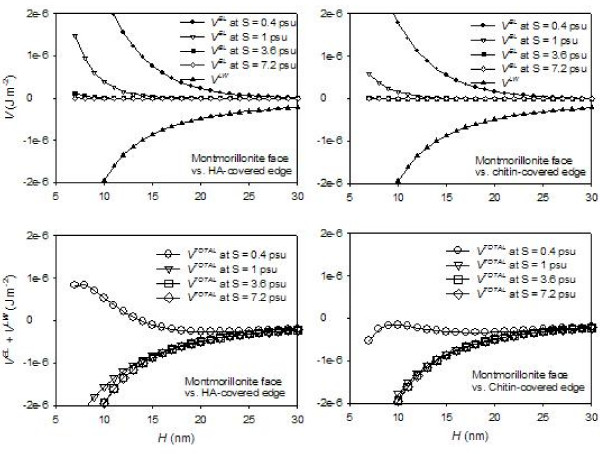
**Surface interaction energy calculated from electrostatic interaction (*V*^*EL*^) (i.e., Equation 1) and Lifshitz-van der Waals interaction (*V*^*LW*^)(i.e., Equation 5)**. The *V*^*EL *^and *V*^*LW *^values are shown individually in the upper figures, whereas the net energy values are shown in the lower figures. The net sum of *V*^*EL *^and *V*^*LW *^remains negative throughout the experimental conditions used to derive the z-potential (i.e., circumneutral pH at salinity values indicated on figure) except for the interaction between face and HA-covered edge at very low salinity (S = 0.4 psu). Net negative surface interaction energy would induce aggregation, whereas our observations yielded very little aggregation for S = 3.6 psu and below. On the other hand, aggregation was observed at S = 7.2 psu.

The values of $V_{ij}^{EL}$ and $V_{ij}^{LW}$, as well as $V_{ij}^{EL}$ + $V_{ij}^{LW}$ were calculated for certain discrete salinity values (i.e., S = 0.3, 1, 3.6, and 7.2 psu) using the ζ-potential values experimentally determined and interpolated at circumneutral pH (Figure 10), and shown in Figure 11. These results show that the sum of electrostatic and Lifshitz-van der Waals forces is constantly negative under the experimental conditions we considered, except for the HA system with very low salinity (S = 0.4 psu). This negative interaction energy would cause particles to approach each other and aggregate. However, montmorillonite did not aggregate in the HA-containing systems at S < 3.6 psu. Meanwhile, aggregation was observed at S = 7.2. Consequently, it is clear that other repulsive forces, such as steric repulsion, are quantitatively important in these systems in order to keep particles well dispersed in suspensions with S = 3.6 psu or less.

**Figure 12:**
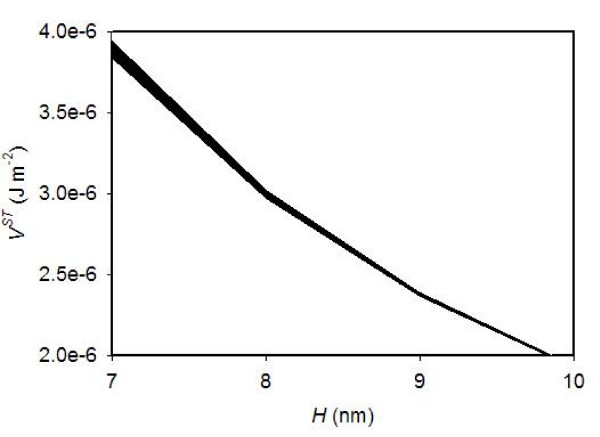
**The estimated range for the magnitude of repulsive forces operating in the montmorillonite-HA suspensions is indicated by the black band**.

The magnitude of steric repulsion can be estimated by comparing the values of *V*^*TOTAL *^(= *V*^*EL *^+ *V*^*LW*^) at S = 3.6 and 7.2 psu. At S = 3.6 psu, the steric repulsion was at least as significant as the negative values of *V*^*TOTAL *^in order to keep the colloids dispersed. On the other hand, at S = 7.2 psu, the steric repulsion was exceeded by the negative values of *V*^*TOTAL*^. It should be noted that the range estimation is conducted under the assumption that the steric repulsion is independent of salinity; thus the range is a rough estimate. In reality, the hydrodynamic diameters of polymers are greater in higher salinity solution due to the polymer unfolding, and thus the steric repulsive forces may be greater in higher salinity solutions [49]. Figure 12 shows the estimated ranges for the magnitude of the additional repulsion (i.e., steric repulsion) *V*^*ST *^for the montmorillonite-HA suspensions determined by bracketing with *V*^*TOTAL *^at S = 3.6 and *V*^*TOTAL *^at S = 7.2 psu. The estimate for montmorillonite-chitin suspensions is not shown but very similar. The *V*^*ST *^values are quantitatively significant, as their values, even though rough estimates, are in the same order of magnitudes as the van der Waals attraction.

## Conclusion

Rivers erode rocks and soils, and carry the mineral particles, especially fine-grained clay mineral particles, down to the estuarine environments. The fate of these particles, whether they are aggregated and settled rapidly or dispersed and remain in suspension for prolonged period of time for further hydrodynamic transport, is significantly influenced by the amount of organic matter that is also suspended in the river water.

Our experimental results indicate that organic macromolecules, which are a major component of many riverine suspensions in general, prevents clay mineral aggregation through (1) augmentation of the electrostatic repulsion between the face and edge surfaces of clay minerals by rapidly adsorbing onto the edge surface and increasing its negative charge; and (2) introduction of steric repulsion. The surface interaction energy calculations indicate that the latter (i.e., steric repulsion) is quantitatively more significant than the augmentation of electrostatic repulsion.

## Competing interests

The authors declare that they have no competing interests.

## Authors' contributions

All authors contributed to the design of the experiments and manuscript draft. YF conceived the study, designed the specific details of the experiments, conducted the interaction energy calculations, and drafted the initial manuscript. JLW carried out the Zetasizer experiments. JK, KJC, and RHB conducted electron microscopy analyses (not reported here) that guided the course of Zetasizer experiments. All authors read and approved the manuscript.
